# In Vitro Host-Cell Susceptibility to Usutu Virus

**DOI:** 10.3201/eid1102.041016

**Published:** 2005-02

**Authors:** Tamás Bakonyi, Helga Lussy, Herbert Weissenböck, Ákos Hornyák, Norbert Nowotny

**Affiliations:** * University of Veterinary Medicine, Vienna, Austria;; † Faculty of Veterinary Science, Budapest, Hungary;; ‡ Central Veterinary Institute, Budapest, Hungary;; § United Arab Emirates University, Al Ain, United Arab Emirates

**Keywords:** Usutu virus, Flaviviridae, cell culture susceptibility, cytopathic effect, immunohistochemistry, dispatch

## Abstract

We investigated the susceptibility to Usutu virus (*Flavivirus*) of 13 permanent cell lines, 3 primary cell cultures, and chicken embryos. Vero, PK-15, and goose embryo fibroblast cells developed cytopathic effects; however, viral multiplication was detected in all mammalian cell types by immunohistochemical tests. Chicken embryo fibroblast cells and chicken embryos were resistant.

Until its emergence in Austria in 2001 (*1*), Usutu virus was regarded as a flavivirus found only in sub-Saharan Africa. The virus was first isolated from *Culex naevei* in South Africa (*2*); later it was detected in other mosquito (*Cx. perfuscus*, *Mansonia africana*, *Coquilletidia aurites*), bird (*Turdus libonyanus*, *Bycanistes fisculator*), and rodent species (*Praomys* sp.) (*3**–**5*). Also, Usutu virus was isolated once from a man with fever and rash (*3*). In Africa, *Culex* mosquitoes and birds are responsible for transmission and circulation of the virus in nature; however, the infection does not cause overt disease in the local host species. Since its introduction to Europe, Usutu virus has shown substantial pathogenicity for several wild bird species and causes severe die-offs, especially in the Eurasian blackbird (*T. merula*) populations. Recurring enzootics have been observed from mid-July to the end of September in the affected areas in the eastern part of Austria within the last 4 years (*6*).

Usutu virus is a member of the Japanese encephalitis virus (JEV) group within the mosquitoborne flaviviruses (*7*). The most important members of the group, West Nile virus (WNV), Murray Valley encephalitis virus (MVEV), St. Louis encephalitis virus (SLEV), and JEV are able to infect a broad spectrum of animal species. These viruses are transmitted by different mosquito species and frequently cause infections in birds (all virus species), rodents (WNV, SLEV), swine (JEV), and horses (WNV, MVEV, SLEV). WNV, SLEV, JEV, and MVEV are human pathogens as well; they may cause epidemics of encephalitis in humans in certain geographic regions.

The classic manner of flavivirus cultivation is intracerebral inoculation of suckling mice or inoculation of embryonated eggs (*8*). A variety of primary cells and established cell lines support the replication of flaviviruses: Green monkey (Vero), hamster (BHK-21), human (SW-13, HeLa), porcine (PS), and mosquito cell lines, as well as primary chicken and duck embryo cells have been used for flavivirus isolation and propagation in routine diagnostic applications. The appearance of cytopathic effects (CPEs), plaque formation, and virus yields greatly vary with the different viruses and host cells.

Since Usutu virus was of minor clinical importance until its emergence in central Europe, its biologic features, host spectrum, and pathogenesis had not previously been studied. With the changes in the clinical appearance of Usutu virus infection in the new environment, and the impact of closely related viruses on human and veterinary health care, the detailed characterization of the virus is of high priority.

## The Study

We investigated the in vitro susceptibility of various cell cultures and embryonated eggs to Usutu virus infection. Human (HeLa), green monkey (Vero), equine (ED), bovine (MDBK), porcine (PK-15), rabbit (RK-13), canine (MDCK, DK), feline (CR), hamster (BHK-21, BF), rat (C6), and turtle (TH1) permanent cell lines, as well as primary horse kidney (EqK), chicken embryo fibroblast (CEF), and goose embryo fibroblast (GEF) cell cultures were tested. Cells were propagated in Earle's minimal essential medium (MEM) (Gibco Invitrogen, Paisley, UK) containing L-glutamine, antimicrobial drugs, and 10% fetal calf serum (FCS). The cells were regularly subcultured by employing standard techniques. To 1-day-old confluent monolayers of the permanent cell lines and primary cell cultures, grown on the surface of chamber slides, the Austrian Usutu virus strain Vienna 2001-blackbird (GenBank accession no. AY453411) was added at a multiplicity of infection (MOI) of 3. The virus was originally isolated in Vero cells in 2001 from the brain homogenate of a blackbird found dead in the area surrounding Vienna. The isolate was propagated twice in Vero cells. The second virus passage was used for the experiments; 50% tissue culture infective dose (TCID_50_) was determined, and aliquots of the virus were stored frozen at –80ºC until used. The virus was added to the cells, which were then incubated at 37°C for 1 h. Thereafter, the inoculum was removed, the cell cultures were washed once with phosphate-buffered saline (PBS), and MEM containing 2% FCS, L-glutamine, and antimicrobial drugs were added. For all cell types, controls were cultivated simultaneously and treated in the same way as the infected cultures with the exception that MEM was used for inoculation. All cell cultures were incubated at 37°C for 3 to 5 days; then the medium was removed and the monolayers were fixed with chilled (–20°C) acetone. The cells were stained with hematoxylin-eosin (HE) and examined microscopically. In parallel, immunohistochemical (IHC) testing was carried out on the cell cultures by using the avidin-biotin complex technique, with a polyclonal antiserum raised in mice against WNV antigens, for which cross-reactivity with Usutu virus had been demonstrated previously (*1*). The number of antigen-positive cells was evaluated microscopically and scored (see Table).

**Table:** Semiquantitative evaluation of the number of Usutu virus antigen–positive cells*

Cell line/culture	IHC result
HeLa (human)	++
Vero (simian)	++
ED (equine)	++
MDBK (bovine)	+
PK-15 (porcine)	++
RK-13 (lapin)	++
MDCK (canine)	++
DK (canine)	(+)
CR (feline)	+
BHK-21 (hamster)	+
BF (hamster)	+
C6 (rat)	+
TH1 (turtle)	++
*EqK* (equine)	++
*CEF* (chicken)	–
*GEF* (goose)	++

*IHC, immunohistochemical; scoring criteria: (+), 1%–5% positive cells; +, 6%–25% positive cells; ++, 26%–50% positive cells. Primary cell cultures are indicated in italics.

Embryonated chicken eggs (strain LSL White, which was derived from the strain White Leghorn), originating from a specified pathogen free (SPF) herd (VALO eggs, Lohmann, Cuxhaven, Germany), were injected into the allantoic sac with 6 x 10^5^ TCID_50_ of Usutu virus at the age of 10 days. The eggs were incubated together with mock-infected controls at 37.5°C for further 4 days and were checked daily by transillumination. On day 4 postinfection the eggs were opened, and the embryos were fixed in 4% buffered formaldehyde solution. Histologic sections were made from paraffin-embedded organs of the embryos, and the slides were analyzed by light microscopy after HE and IHC staining, respectively, as described above.

**Figure 1:**
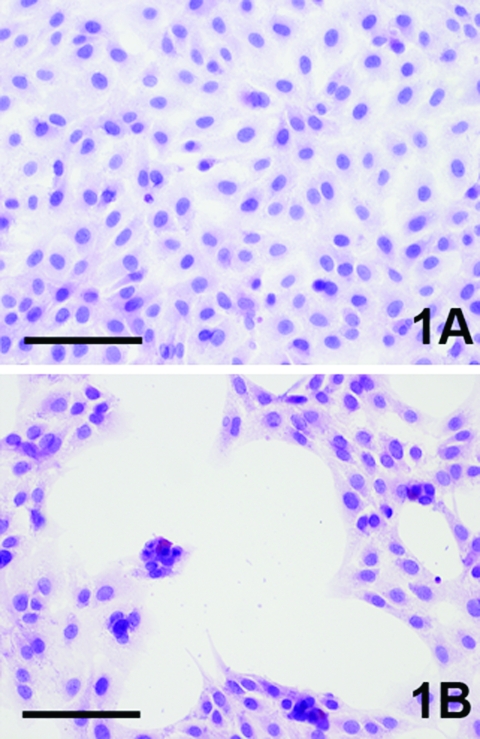
Cytopathic effect (CPE) of Vero cells caused by Usutu virus infection, 4 days postinfection (hematoxylin-eosin staining). A) Uninfected control, B) Usutu virus infected. bar = 100 µm.

**Figure 2:**
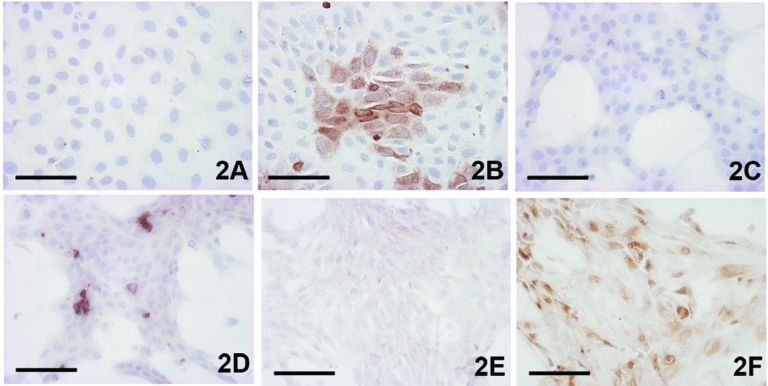
Demonstration of Usutu virus antigen 3 days postinfection. Immunohistochemical (IHC) tests were performed by using a polyclonal antibody to West Nile virus, which cross-reacts with Usutu virus. A) Vero control; B) Vero infected; C) CR [define] (feline) control; D) CR infected; E) goose embryo fibroblast (GEF) [define] control; F) GEF infected; A,B) bar = 50 µm; C–F) bar = 100 µm. IHC staining.

Three to 4 days after inoculation, pronounced CPEs were observed in Usutu virus–infected Vero and PK-15 cell cultures as well as in GEF cells. The first foci of cell rounding and subsequent shrinkage of the cells were observed on day 2 or day 3 postinfection, when groups of 4 to 8 cells, but also single cells, showed rounding and degeneration; within 1 day the affected cells lost their adherence to the bottom of the flask and floated in the medium. Within a further 2 days, 90%–100% of the cells exhibited CPE. Typical Usutu virus CPE is shown in HE-stained Vero cells in Figure 1. The mock-infected Vero, PK-15, and GEF control cell cultures did not show any CPE. The other investigated cell types inoculated with Usutu virus did not develop visible CPE within a period of 5 days, and they were also negative by microscopy after HE staining. However, by IHC with cross-reactive WNV-antiserum, focal virus multiplication was detected in all cell cultures, independent of animal species and tissue type, except chicken embryo fibroblast cells (Figure 2). The percentage of Usutu virus antigen–positive cells varied from ≈1% (DK) to 50% (GEF) (Table). In the case of HeLa cells, different clones adapted to the propagation of human rhinoviruses (HeLa Rhino) and herpes simplex viruses (HeLa HSV), respectively, were also tested, but they gave the same results as the commonly used (ATCC) HeLa cells by HE and IHC staining. The mock-infected control cell cultures were clearly negative in each case.

The Usutu virus–infected chicken embryos did not show any lesions when investigated by gross and histopathologic examination after 4 days of incubation and were negative by IHC as well. To rule out the slight possibility that the Usutu virus strain used for inoculation underwent a change in cell tropism during the 2 passages in Vero cells, CEF, Vero, PK-15, MDCK, and DK cells, as well as embryonated chicken eggs, were reinfected with the original Usutu virus isolate (before passaging); the results were identical to the results obtained with Usutu virus passaged twice before use.

## Conclusions

The appearance of CPE in flavivirus-infected cell cultures depends on the virus and host cell type, as well as on MOI levels and incubation time employed (*8*). In many cases, the presence and multiplication of flaviviruses do not inhibit significantly the host cell macromolecular synthesis, resulting in noncytopathic persistent infections (*9**,**10*). Pathogenesis and virulence of flaviviruses are influenced in vivo by several virus- and host-dependent factors, including the role of defective interfering particles, viral receptors, neurovirulence, immune-response (e.g., antibody-dependent enhancement), and host resistance genes (*8*). Although some of these processes are not yet fully understood, the basic requisite of any pathogenic effect is the host susceptibility to the virus infection. This study demonstrates that Usutu virus can infect cell cultures of various tissue types derived from a wide variety of animal species, including cell lines of human origin. Since only Vero, PK-15, and GEF cells develop CPE after Usutu virus infection, these cell lines and cell culture are the most appropriate ones for diagnostic purposes (e.g., virus isolation, plaque reduction neutralization test). As demonstrated by IHC, considerable differences have been found in the susceptibility of the various cell lines and cultures to Usutu virus infection and in the extent of spread of the infection; even cell lines derived from the same animal species and organ varied significantly in their susceptibility to Usutu virus infection, e.g., MDCK cells strongly support Usutu virus multiplication, while DK cells are far less susceptible. Both of these cell lines, however, have been derived from dog kidneys. On the other hand, the differences between the 2 canine kidney cell lines might also be the consequence of different random mutations (e.g., in genes of the interferon or other innate defense systems) that allowed the cells to immortalize. Since in Austria, Usutu virus infects wild birds and causes high death rates, especially in blackbirds, one would think that birds are most susceptible hosts for the virus. Therefore, the finding that both the chicken embryo fibroblast monolayers and the chicken embryos are apparently resistant to Usutu virus infection was unexpected. Usutu virus, however, is not the only flavivirus with such contradiction in host spectrum. Ilheus virus, a South American mosquitoborne flavivirus belonging to the Ntaya virus group (*7*), also naturally affects wild birds and produces plaques in primary rhesus kidney cells and various established cell lines (Vero, PS, BHK-21, and LLC-MK2), but not in avian cells (*8*). Preliminary results of our chicken experiments with Usutu virus also support that idea that the domestic chicken is resistant to the infection, even when young. Further investigations involving different bird and mammal species will be necessary to show the most important host species, natural reservoirs, and vectors of Usutu virus and to estimate its epidemiologic impact and possible threat to domesticated animals and to the human population.
